# Editorial: Diagnostic burdens in high myopia

**DOI:** 10.3389/fopht.2026.1870549

**Published:** 2026-05-22

**Authors:** Hitomi Saito, Linda Zangwill, Jasmin Rezapour, Hongli Yang

**Affiliations:** 1 The University of Tokyo, Graduate School of Medicine, Department of Ophthalmology, Tokyo, Japan; 2 University of California San Diego, San Diego, CA, United States; 3 University Medical Center Mainz, Mainz, Germany; 4 Legacy Devers Eye Institute, Portland, OR, United States

**Keywords:** artificial intelligence, axial length, corneal curvature, diagnosis, epidemiology, glaucoma, myopia, optical coherence tomography

High myopia is no longer a niche refractive condition encountered only in selected ophthalmic subspecialty clinics. It has become a major and expanding public health concern, driven by the global rise in myopia, particularly high myopia and accompanied by an increasing burden of sight-threatening ocular complications. The diagnostic challenge in high myopia lies not simply in measuring refractive error or axial length, but in recognizing when elongation of the eye has led to structural changes that often mimic other ocular pathologies like glaucoma.

The Research Topic *“Diagnostic Burdens in High Myopia”* was organized to address this complexity. The included articles approach the problem from complementary perspectives: measurement reliability, epidemiology, structural risk stratification, new axial myopia metrics, glaucoma detection, and the role of artificial intelligence. Together, they highlight that high myopia cannot be adequately managed through a single diagnostic parameter. Instead, it requires careful integration of refractive, biometric, structural, functional, and increasingly, computational information.

A fundamental diagnostic burden begins with measurement itself. Qu et al. compared corneal curvature and eccentricity measurements obtained using four different devices in myopic eyes. Their findings showed that central corneal curvature measurements had low agreement across instruments and should not be used interchangeably in clinical practice, whereas flat eccentricity measurements showed better comparability among selected topographers. This study is particularly relevant to myopia management, including orthokeratology, where small differences in corneal measurements may influence lens selection and fitting. More broadly, it reminds us that diagnostic pathways in myopia depend on device-specific measurements, and that apparent clinical differences may sometimes reflect measurement variability rather than biological change.

The challenge of defining axial myopia is further explored by Ren and Chu, who proposed “Myopic Strain” as a normalized metric for assessing axial myopia. Axial length remains the standard measure of ocular elongation, but it does not fully distinguish between the eye’s optical focal distance and excessive elongation beyond emmetropic requirements. By defining Myopic Strain as the ratio of retinal defocus distance to the eye’s focal length, the authors attempted to provide a more size-normalized and conceptually interpretable index. Their analysis showed that Myopic Strain correlated strongly with spherical equivalent refractive error and was also associated with biomechanical markers. Although further validation is needed, this work is valuable because it challenges the field to move beyond axial length alone and toward metrics that better capture the optical and biomechanical meaning of elongation.

Epidemiological context is essential because diagnostic burden is also a health-system burden. Magaña-Lizárraga et al. provided a large nationwide clinic-based analysis of myopia in Mexican outpatients. In more than 3.5 million records, the authors reported an age-standardized prevalence of total myopia of 44.44% and high myopia of 1.12%. Although high myopia was relatively uncommon compared with reports from some East and Southeast Asian populations, its increase from childhood to early adulthood underscores the need for early detection and longitudinal monitoring. The study also identified geographic variation and a strong association between astigmatism severity and myopia risk. These findings expand the geographic scope of high-myopia epidemiology and emphasize that even modest prevalence rates can translate into substantial future burdens when applied to large populations.

While epidemiological studies quantify risk at the population level, clinicians must identify which individual eyes already show structural damage. Li et al. addressed this by examining the clinical spectrum and prevalence associations of chorioretinal damage in high myopia. In a retrospective cross-sectional cohort of 1,420 patients and 2,610 eyes, chorioretinal damage was documented in 40.0% of eyes. Diffuse atrophy, foveoschisis, and patchy atrophy were the most common lesions. Older age, longer axial length, more myopic spherical equivalent, and thinner choroid were independently associated with pathology. Importantly, the study used the atrophy-traction-neovascularization (ATN) classification and multimodal imaging, demonstrating how standardized phenotyping can support risk-stratified surveillance. This is a key step toward translating structural complexity into clinically actionable monitoring strategies.

One of the most difficult diagnostic problems in high myopia is glaucoma detection. Myopic optic discs may be tilted, enlarged, and surrounded by peripapillary atrophy, while retinal nerve fiber layer thickness measurements may be difficult to interpret. Bowd et al. examined whether deep learning autoencoder-based regions of interest derived from swept-source OCT texture en face images could improve glaucoma detection in myopic eyes. Their dual-autoencoder model, trained to capture both healthy and glaucomatous patterns, outperformed global RNFL thickness, texture en face analysis alone, and a single autoencoder model. This approach is clinically meaningful because it attempts to distinguish glaucomatous damage from myopia-related structural variation, one of the central diagnostic dilemmas in myopic glaucoma.

Finally, Zhang et al. provided a broad review of diagnostic challenges in high myopia and the growing role of artificial intelligence. The review synthesized the diagnostic difficulties associated with myopic maculopathy, choroidal neovascularization, retinal detachment, and glaucoma, emphasizing that overlapping and atypical presentations can delay timely intervention. It also highlighted the value of OCT, ultra-widefield imaging, fluorescein angiography, OCT angiography, and AI-based systems for detecting high-myopia-related complications. The review points toward a future in which multimodal imaging, biomarkers, and AI-driven prediction models are combined to support personalized surveillance and earlier intervention.

Taken together, the articles in this Research Topic show that the diagnostic burdens of high myopia are multidimensional. They arise from measurement variability, inconsistent definitions, population heterogeneity, overlapping phenotypes, and the limitations of conventional structural metrics. They also arise from the fact that high myopia is not a single disease state, but a spectrum ranging from refractive elongation to complex degenerative pathology. The field therefore needs not only better instruments, but better frameworks: standardized definitions, longitudinal data, multimodal imaging protocols, and validated models that can separate benign myopic anatomy from early disease.

